# A new hybrid risk assessment method based on Fine-Kinney and ANFIS methods for evaluation spatial risks in nursing homes

**DOI:** 10.1016/j.heliyon.2022.e11028

**Published:** 2022-10-13

**Authors:** Seda Hatice Gökler, Didem Yılmaz, Zerrin Funda Ürük, Semra Boran

**Affiliations:** a Department of Industrial Engineering, Kahramanmaras Sutcu Imam University, Turkey; b Department of Industrial Engineering, Istanbul Gelisim University, Turkey; c Department of Interior Design, Faculty of Art and Design, Istanbul Nisantasi University, Turkey; d Department of Industrial Engineering, Sakarya University, Turkey

**Keywords:** Fine-kinney risk assessment method, ANFIS method, Nursing home, Spatial risks

## Abstract

Today, as the elderly population in the world increases, the increase in those living in nursing homes causes their problems to be even more important. Spatial hazards cause injury and death most of the time, therefore should be evaluated risks then corrective and preventive actions should be implemented. Fine-Kinney is one of the most widely used risk assessment methods, but it has some shortcomings. One of them is that risk factors such as probability, frequency, and severity are accepted as equally important, but they can have different importance weights in real-life applications. Another is that experts assess the risk magnitudes using their opinions, who usually tend to use linguistic expressions instead of crisp numbers, in incomplete information and uncertain situations. The last is that the experts' experiences are not effectively incorporated into the automation of the risk assessment. The adaptive neuro-fuzzy inference system (ANFIS) method, which is a machine learning method, can overcome all these shortcomings.

In this study, a novel hybrid risk assessment method based on Fine-Kinney and ANFIS is developed to predict the class of a new occurring risk. The hybrid method was applied to nursing homes located in Turkey. The risk classes predicted with the hybrid method were compared to ones found in the traditional Fine-Kinney method. It was determined that the prediction accuracy and Fleiss kappa value of the new hybrid method were 95.745% and 0.929 respectively. Thus, the hybrid method can be used instead of the traditional Fine-Kinney method to determine the class of a new risk, because it does not require a large number of experts and provides a faster assessment.

## 1 Introductıon

The world population is rapidly aging, especially in developed countries. 9.8% of the world's population was the elderly population in 2021 [1]. In Turkey, the average age of the population started to increase similarly. While the elderly population in Turkey was 5 million 327 thousand 736 people in 2010, it increased by 49% in the last ten years and reached 7 million 953 thousand 555 people in 2020. According to population projections, it is predicted that the proportion of the elderly population would be 11% in 2025, 12.9% in 2030, 16.3% in 2040, 22.6% in 2060, and 25.6% in 2080 [2]. In general, the increase in the elderly population in the world causes their problems to be more important today.

Nursing homes have started to replace family support for the elderly who have difficulty maintaining their lives alone. Demand for nursing homes also increases while the elderly population increases. Nursing homes are residential social service establishments established to protect and care for elderly people aged 65 and over in a peaceful environment and to meet their social and psychological needs [3]. However, elderly people may face spatial risks due to unsuitable or non-ergonomic area characteristics while performing their daily functions. Risks need to be identified to develop effective strategies to reduce or eliminate the risks and thus ensure the quality of life for residents in nursing homes. Early identification of risks provides for the earlier implementation of prevention activities, thus also reducing the consequences of injury and death. Eliminating spatial hazards and making ergonomic regulations in the nursing home increases the safety and mobilization of the elderly.

In the literature, studies have been achieved to evaluate the relationship between environmental factors and risk factors, especially the falls in the homes where the elderly spend almost all of their time. Home hazards are accepted as one of the most important factors contributing to the risk of falls [4, 5, 6, 7]. Carter et al. (2000) studied whether socio-demographic characteristics, medication use, environmental hazards in the home, and other potential risk factors were associated with all accidents and falls [8]. Lord et al. (2006) examined the role that environmental hazards play in increasing the risk of falls and evaluated the efficacy of environmental interventions to reduce falls [6]. Lök and Akın (2013) explored the relationship between the risk factors in the home conditions and the falling of the elderly [9]. Chakpitak et al. (2015) investigated whether cluttered home conditions are a significant risk of falling in older people [10]. Romli et al. (2018) aimed to identify standardized instruments for evaluating home hazards related to falls [11].

A study on improving the quality of life in the nursing home was achieved by Eijkelenboom et al. (2017) [12]. They investigated only which architectural factors contribute to a sense of home and how these can be implemented in the design guidelines but not considered spatial risks in the home.

In this study, unlike the studies in the literature, the spatial hazards that the elderly living in the nursing home may be exposed to were evaluated by applying a risk assessment method. Using the risk assessment method provides to prevent to not ignore significant risks and not using unnecessary effort and resources for insignificant risks. It is aimed to evaluate the spatial risks by the Fine-Kinney method, which is one of the most common risk assessment methods.

The Fine-Kinney method assesses risks according to probability, frequency, and severity that are risk factors and prioritizes based on the risk score that is a product of three ones. There are scale tables of each factor that includes the score and its definition. Risk assessment experts use the tables to convert existing information into numbers.

The Fine-Kinney method was applied to assess risks in different areas, such as ballast tank maintenance [13], wind turbine construction and operation [14], and railway transportation systems [15].

But traditional Fine-Kinney method has some shortcomings. One of them is that experts have to make assessments with incomplete information usually, and in this case, they need to use their knowledge and experience. Accordingly, the results of the risk assessment may vary according to the knowledge, experience, and initiatives of the safety experts. For example, in assessing risk, one expert might assign a small value to the severity risk factor, while another expert might assign a much larger value. Another is that experts usually tend to use linguistic expressions instead of crisp numbers, then it can be difficult to rate risk by crisp numbers in actual conditions. Another is that risk factors are accepted as equally important. However, they can be considered to be different from each other in real-life applications. The last is that the expert's experience is not effectively incorporated into the automation of the risk assessment.

The methods such as AHP [16], fuzzy AHP and fuzzy VIKOR [17], Pythagorean fuzzy AHP [18], k-means [19], and COPSOQ II questionnaire [20], are integrated into the Fine-Kinney method to overcome its shortcomings. Fuzzy logic provides to transform linguistic risk information into quantitative risk rating information. Fuzzy logic can provide a more flexible and effective means to express complex and uncertain risk evaluation information. But the shortcoming of fuzzy logic is that it requires users to design the if-then rules and doesn't have the learning capability of machine learning methods.

Machine learning techniques can add much to the risk assessment field [21]. The adaptive neuro-fuzzy inference system (ANFIS) method which is a combination of Artificial Neural Network (ANN) and fuzzy logic methods, is a machine learning method. It includes the positive features of both methods such as parallel computation and learning ability of ANN, and inference system of fuzzy logic. ANFIS can be used to overcome its shortcomings in the Fine-Kinney method. ANFIS method was applied in hazards evaluation, existing in very different areas. Lo et al. (2009) used ANFIS to predict the pre-evacuation behavior of people under fire situations that are of prime importance to fire safety design in buildings, especially for complex and ultra-high rise buildings [22]. Wang et al. (2012) proposed an ANFIS model optimized with an ant colony search method that is used to predict the high operational risks of the operator [23]. The model been has employed under a series of process control tasks on a simulated software platform of automation-enhanced Cabin Air Management System. Ebrat and Ghodsi (2014) used the ANFIS method to determine the priority of risk factors of construction projects and to predict risk with high accuracy [24]. Fragiadakis et al (2014) have applied ANFIS to examine the effect of working conditions on the occupational injury of accidents while ship repair [25]. Liu and Chen (2017) used the ANFIS method to predict real-time crash risk occurrence on the expressway [26]. Zhou et al. (2019) applied ANFIS to predict the risk of near-miss incidents during tanker shipping voyages [27]. They analyzed causal factors in terms of direct contributory factors, indirect contributory factors, and root contributory factors to the near-miss incidents and defined risk control measures to improve safety during tanker shipping. Jahangiri et al (2019) combined the ANFIS method with a safety inspection checklist to identify risk factors and predict the risk of falling from the scaffold on construction sites [28]. Omidia et al. (2019) used the ANFIS model to predict patient safety grades in healthcare organizations [29].

A study using Fine Kinney and ANFIS methods was achieved by Baç and Ekmekçi (2020) [30]. They evaluated the psychosocial risks of maintenance workers in their study. However, in the study, the two methods were not integrated, the data obtained from the COPSOQII questionnaire was used as the input in each method.

This study, unlike studies in the literature, it is aimed to develop a hybrid risk class prediction method by integrating a Fine Kinney risk assessment method and the ANFIS method.

The developed hybrid method was applied to assess the spatial risks of 29 selected nursing homes in Istanbul, which is the city with the largest number of nursing homes in Turkey. The risk classes predicted with the new hybrid method and found with the traditional Fine Kinney method are compared. It has been determined that the developed hybrid method can predict risk classes with 95.745% accuracy. So, the new hybrid risk assessment method based on Fine-Kinney and ANFIS methods can be used instead of the traditional Fine-Kinney method. Thus, more appropriate architectural and ergonomic solutions that facilitate the daily life activities of the elderly and increase their quality of life can be fast developed.

This study contributes to the literature as follows:

• ANFIS method is integrated into the Fine-Kinney method to predict a new occurring risk's class
• Spatial hazards in the nursing home are analysed by using a risk assessment method

The rest of this article is organized as follows. Methods are explained in section 2. The proposed new integrated method is introduced in section 3. Results are discussed in section 4. Finally, the conclusion is summarized in section 5.

## 2 Methods

### 2.1 Fine-Kinney risk assessment method

The Fine-Kinney risk assessment method is a useful quantitative technique for assessing risks [31]. Each risk is assessed considering probability (P), frequency (F), and severity (S) which are risk factors. Probability is the possibility that the risk (hazard) will occur over time. Frequency is the frequency of exposure to danger. Severity represents the magnitude of the damage it causes to people and/or the environment when a hazard occurs. The numerical values of the factors are determined from the standard tables structured (Table 1). The values of the probability risk factor range from 0.1 to 10 whereas frequency values range between 0.5 and 10. Severity can take a value between 1 and 100. The scores of risk factors usually are obtained by experts. They consider past data and use their observations in the workplace while assessing risk factors. The risk score (R) is determined by multiplying the probability, frequency, and severity score (Eq. 1).

(1) Risk=Probability×Frequency×Severity

**Table 1:** Risk factors.

(P)	Definition	(F)	Definition	(S)	Definition
0.1	Virtually impossible	0.5	Very rare	1	Injury without work capacity loss –noticeable
0.2	Practically impossible	1	Rare	3	Injury with loss of work capacity -important
0.5	Plausible, but unlikely - Conceivable but very unlikely	2	Monthly-unusual	7	Important damage-serious
1	Improbable, but possible at boundary conditions - Only remotely possible	3	Occasional	15	Permanent damage-very serious
3	Unusual, but possible	6	Regular frequent	40	One fatalities-disaster
6	Possible - Quite possible	10	Permanent-continuous	100	Several fatalities- Catastrophe
10	Predictable- Might well be expected				

Risks are defined in five classes such as very high risk, high risk, substantial risk, possible risk, and acceptable risk, according to their scores (Table 2). 'A' class risk requires immediate prevention on the other hand the 'E' class risk is less risky than the other classes.

**Table 2:** Risk scores and classes.

Risk Score (R)	Risk Level	Risk Class
400 ≤ R	Very high risk (Activity cessation)	A
200 ≤ R < 400	High risk (Immediate improvement)	B
70 ≤ R < 200	Substantial risk (Measures to be taken)	C
20 ≤ R < 70	Possible risk (Monitoring)	D
R < 20	Acceptable risk (No measure required)	E

### 2.2 ANFIS method

The ANFIS method was developed by Jang (1993) [32], which is a combination of ANN and fuzzy logic methods. ANFIS includes the advantages of both methods. ANN method, which has a hybrid learning algorithm consisting of both back propagation learning and the least-squares method, allows to classify and identify patterns. Its training process is data-based. Fuzzy logic contains if-then rules called fuzzy inference systems (FIS) that are used in the training process of the ANN model. The input and output relationships of the model are explained by rules determined from expert experience. ANN model is self-learning with the linguistic expression function of FIS. Thus, the ANFIS model has the advantage of having both numerical and linguistic knowledge [33]. The method enables fast and accurate learning; provides excellent explanation facilities the uncertain situations through fuzzy rules [34]. The feature that makes the ANFIS method superior to ANN is that it allows the user to add their own rules.

Mamdani and Sugeno developed FISs, the most commonly used. In the literature, the fuzzy- Sugeno method is more preferred than the fuzzy Mamdani method. Sugeno FIS has fuzzy inputs as Mamdani but it does not need the defuzzification step. The Sugeno FIS outputs membership functions (MF) are linear or constant, so Sugeno outputs are crisp values [35]. Thus Fuzzy-Sugeno is to reduce the number of rules required by the Mamdani model [36]. The basic rule structure for two inputs and one output can be defined as follows for the first-order Sugeno model (Eq. 2).

(2) Rule 1: If *x* is A1 and *y* is B1*,* then f1*=*p1x*+*q1y*+*r1

**Figure 1:**
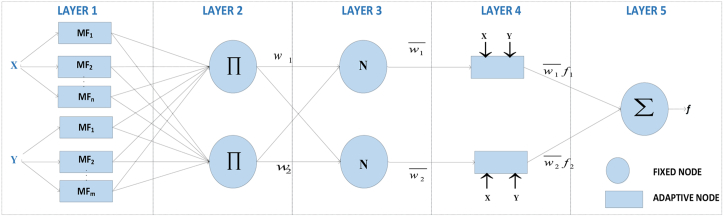
The architecture of the ANFIS model.

Rule 2: If *x* is A2 and *y* is B2*,* then f2*=*p2x*+*q2y*+*r2

where x and y express inputs and f represents output; $A_{i}$ and $B_{i}$ are the membership functions of each input x and y; $p_{i}$, $q_{i}$, and $r_{i}$ are linear output parameters. ANFIS model for two inputs and one output and two rules is shown in Figure 1.

The architecture of ANFIS consists of five layers of which neurons in the same layer are contained in the same function family. The layers are explained as follows.

*Layer 1 (Fuzzification Layer).* Every node *i* in this layer is an adaptive node, which can be any parameterized by a membership function (MF), such as Triangle, Trapezoidal, Gaussian, or generalized Bell function. The outputs of the layer (Os) are the fuzzy membership grade of the inputs, which are represented as follows (Eq.3):

(3) $$O_{1,i}=\mu_{A_{i}}\left(x\right),i=1,2$$

$$O_{1,i}=\mu_{B_{i}-2}\left(y\right),i=3,4$$

where x and y are the inputs to node *i*; A and B are the linguistic labels (small, medium, high, etc.) associated with the node function. Here, $\mu_{A_{i}}\left(x\right)$ and $\mu_{B_{i}-2}\left(y\right)$ are adopted linear or nonlinear fuzzy membership functions.

*Layer 2 (Rule Layer).* Every node in the second layer is a fixed node representing the product ∏ to calculate the firing strength of a rule. The fuzzy AND operator is used to fuzzify the inputs. The outputs of this layer which are called firing strength of rules can be represented as shown in Eq. (4)

(4) $$O_{2,i}=w_{i}=\mu_{A_{i}}\left(x\right)\mu_{B_{i}}\left(y\right),i=1,2$$

The number of rules generated is equal to *m*^*n*^, where m is the number of MFs in each input variable and n is the total number of inputs to the ANFIS model.

*Layer 3 (Normalization Layer).* Every node is fixed (circle) and labeled as N. Each node is normalized by dividing the *i*th rule's firing strength by the sum of all rules' firing strength. Outputs of this layer that are called normalized firing strength can be represented as (Eq.5)

(5) $$O_{3,i}={\bar{w}}_{i}=\frac{w_{i}}{\begin{array}{cc} w_{1}+ & w_{2} \end{array}}\;,i=1,2\;$$

where ${\bar{w}}_{i}$ is defined as the normalized firing strength of a rule.

*Layer 4 (Defuzzification Layer).* Every node is adaptive with a node function. The output of the fourth layer is the product of normalized firing strength and first-order polynomial and is represented as follows (Eq.6)

(6) $$O_{4,i}={\bar{w}}_{i}\;f_{i}={\bar{w}}_{i}\;\left(\begin{array}{ccc} p_{i}x+ & q_{i}y+ & r_{i} \end{array}\right),i=1,2$$

Where $p_{i}$ , $q_{i}$, and $r_{i}$ are the consequent parameters.

*Layer 5 (Output Layer).* The single node in the last layer is a fixed node. The layer represents the overall output of the ANFIS model which is the summation of outputs of all rules. The overall output can be represented as follows (Eq. 7).

(7) $$O_{5,i}=\sum_{i}\bar{w}f_{i}=\frac{\sum_{i=1}^{n}\bar{w_{i}}f_{i}}{\sum_{i=1}^{n}w_{i}}$$

#### 2.2.1 Performance criteria

The performance criteria are used to determine the prediction accuracy of machine learning methods. Overall accuracy is calculated according to a confusion matrix that is based on the user's accuracy and the producer's accuracy. The equation of overall accuracy is as follows (Eq.8).

(8) $$Overall\;accuracy=\frac{total\;number\;of\;correct\;classified}{total\;number\;of\;risks\;class}$$

In this study, the "Fleiss' kappa coefficient" performance criterion (Eq. 9) was also used since the number of experts making evaluations was more than two [37].

(9) $$K=\frac{n\sum_{i=1}^{p}x_{ii}-\sum_{i=1}^{p}\left(x_{i+}x_{+i}\right)}{n^{2-}\sum_{i=1}^{p}\left(x_{i+}x_{+i}\right)}$$

where, n = total number of risk, p = number of class, $\sum x_{ii}$ = total number elements of confusion matrix, $\sum x_{i+}=$ sum of row i, $\sum x_{+i}=$ sum of column i.

In the classification defined by Fleiss (1981), a Kappa value of 0.75 and above indicates excellent, between 0.40-0.75 medium-good, below 0.40 weak prediction accuracy [38].

## 3 A proposed hybrid risk assessment method

It is aimed develop hybrid risk class prediction method that integrates ANFIS method to the Fine Kinney risk assessment method In the novel hybrid method, the Fine Kinney method provides to identify potential spatial risk in the nursing home, while the ANFIS method optimizes the complex relationship among risk factors and then predicts the risk magnitude in the nursing home. The hybrid method achieves to identify and assess the most significant risk class that preventive and corrective measures have a positive impact on safety, and help prevent an accident from occurring in the nursing home. The need for expert judgments for risk assessment is eliminated, and the new risk values can be estimated without the need for experts. Because the ANFIS model learned from data obtained with the Fine-Kinney risk method. ANFIS also lets to the risk factors having significant weights. The severity risk factor was considered more important than other factors such as occurrence and detection in this study. Thus, risks with high severity risk factors are eliminated primarily, regardless of risk scores.

**Figure 2:**
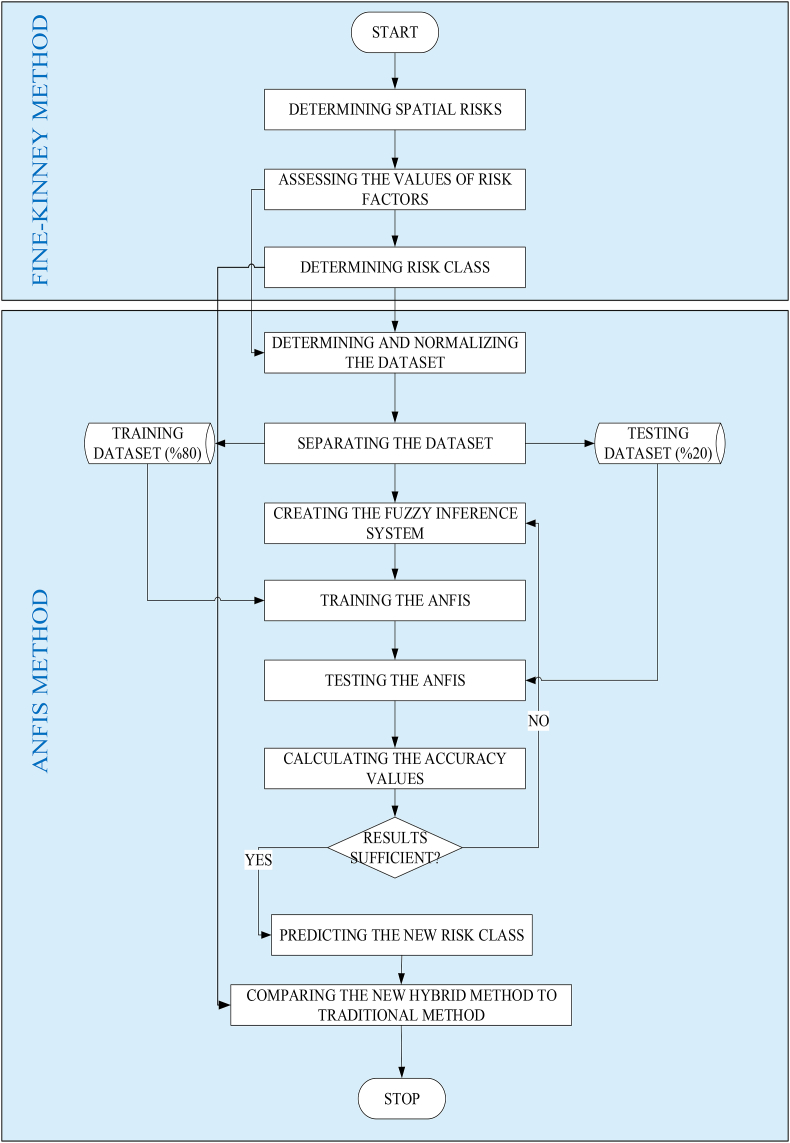
The proposed novel hybrid risk assessment method.

The developed novel hybrid risk assessment method consists of two phases (Figure 2).

In the first phase, with the Fine-Kinney method, the risks are determined, each risk is scored according to the risk factors and the risk class is determined according to the magnitude of the risks.

In the second phase, the ANFIS model is created and trained with the data of the Fine-Kinney method. The probability, frequency, and severity scores of risks are inputs while risk class is the output of the ANFIS model.

## 4 Results and discussion

119 of a total of 439 private and public nursing homes in Turkey are in Istanbul. The method was applied in 29 selected nursing homes in Istanbul. It is seen that the sample size is sufficient [39]. The age range of people residing in nursing homes was 68.1–79.7 and the average was 74. The method was implemented by a team consisting of 3 who were selected among experts who have an 'Occupational Health and Safety' certificate and have at least ten years of experience in spatial risk.

The risks were identified after examining the spatial plans and areas of the nursing homes by experts. Experts also took the opinions of the employees as a basis. A total of 47 hazards were identified in nursing homes. The areas with hazards in the home were divided into 5 classes: stairs and elevators, corridors, rooms, WC-bath, and social areas. Experts assigned scores for probability, frequency, and severity based on Table 1. The score of each risk factor was determined by the consensus of experts. The class of each risk was determined according to the risk score by using Table 2. Analysis results were written in the relevant columns in Table 3. The distribution of the risks of each area according to the class is given in Table 4. As it can be seen from Table 4, areas such as the bathroom, room and stairs, and elevator where the elderly stay alone are at higher risk. Of the 47 risks, 28 are class A, 6 are class B, 5 are class C, 3 are class D and 5 are class E.

**Table 3:** Risk values found with traditional fine-kinney method and ANFIS- fine-kinney approach.

Hazard	Risk	S	P	F	Risk score	Preventive and corrective actions	Fine-Kinney Risk Class	ANFIS Risk Class
BATHROOM								
B1. Using slippery material on the floor	Injury or death by falling due to slipped foot	100	10	10	10000	Selecting the floor material from non-slip	A	A
B2. Presence of abrasions and breaks in the floor	Death or serious injury from tripping on the floor	100	10	10	10000	Repairing the worn areas on the floor	A	A
B3. Existence of thresholds at transitions	Falling as a result of tripping over the threshold and difficulty in responding in an emergency	40	6	10	2400	Removing the thresholds to prevent foot tripping and making them demountable where they need to be used	A	A
B4. The door dimensions do not comply with standards	Difficulty in the passing of a wheelchair, and especially with a stretcher when necessary	7	6	6	252	Making the bathroom doors by the size of the stretcher and wheelchair	B	B
B5. The emergency call button is not available	Having difficulty getting help	100	3	3	900	Installing an emergency call button	A	A
B6. Lighting does not exist correctly and adequately	Injury from striking objects or falling by tripping over objects on the floor	100	10	6	6000	Installing appropriate quality and quantity lighting fixtures	A	A
B7. The grab bars not mounting according to ergonomic measures	Injury from falling due to not being able to hold on to bars	40	3	3	360	Mounting of grab bars on bathroom walls by ergonomic dimensions	B	B
B8. Not to be grab bars in the bathroom	Serious injury from falling in case of loss of balance	40	10	6	2400	Mounting grab bars on both walls of the bathroom	A	A
B9. Incorrect being of height measurements of the toilet seat	Staggering due to difficulty sitting	7	1	2	14	Mounting the toilet seat at a height suitable for the elderly	E	E
CORRIDOR								
C1. Existence of thresholds at transitions to rooms, bathrooms, and other areas	Falling as a result of tripping over the threshold	40	6	6	1440	Removing the thresholds to prevent foot tripping and making them demountable where they need to be used	A	A
C2. Presence of abrasions and breaks in the floor	Injury from falling due to foot tripping	40	10	10	4000	Using suitable flooring materials	A	A
C3. Having no bars on the sides of the corridor	Injury by falling due to not being able to hold on in case of loss of balance	7	3	3	63	Correctly installing the grab bars on both sides of the corridors	D	D
C4. The grab bars are not mounted according to ergonomic measurement	Injury by falling due to not being able to hold on in case of loss of balance	7	1	3	21	Mounting the grab bars on the two side walls of the corridor by ergonomic measurement	D	E
C5. Corridor width is not designed to suit wheelchairs, stretchers, and elderly pass	Difficulty in the passing of a wheelchair, and especially with a stretcher when necessary	15	1	10	150	Designing the corridor width by wheelchair and stretcher passage	C	B
C6. The direction signs are not being along the corridors	Difficulty evacuation in emergencies	7	1	2	14	Determining the route to be used in emergencies and ensuring that direction signs are in appropriate places and numbers along the corridor	E	E
C7. The emergency call button is not available	Having difficulty getting help	15	6	6	540	Installing an emergency call button	A	A
C8. Emergency exit, fire escape, and fire tubes are not available	Emergency response and evacuation difficulties, death and serious injury	100	0.2	1	20	Emergency exit stairs are being in places that elderly people can easily reach and fire extinguishers are in the right places along the corridor	D	D
C9. Lighting does not exist correctly and adequately	Injury from falling by tripping over objects on the floor	40	6	10	2400	Installing appropriate quality and quantity lighting fixtures	A	A
ROOM								
R1. Presence of abrasions and breaks in the floor	Injury from falling due to foot tripping	40	6	10	2400	Making the floor non-slip and suitable material	A	A
R2. Excessive wear on the floor	Injury from falling due to foot tripping	40	6	10	2400	Repairing the worn areas, making the floor material from non-slip and suitable material on the floor	A	A
R3. Using of non-ergonomic furniture in rooms	Injuring from hitting the corners and edges of furniture	15	3	6	270	Making the furniture used in the room by ergonomic and elderly or disabled elderly standards	B	B
R4. The furniture used in the place is made from material that is not suitable for health and the existence of manufacturing defect	Injuring from hitting the corners and edges of furniture	15	3	6	270	Replacing the furniture used in the room with ones made of healthy materials and without manufacturing defects	B	B
R5. Lighting does not exist correctly and adequately	Injury from striking objects or falling by tripping over objects on the floor	40	6	6	2400	Installing appropriate quality and quantity lighting fixtures	A	A
R6. Existence of threshold at transitions to room and bathroom	Falling as a result of tripping over the threshold and difficulty in responding in an emergency	40	6	6	2400	Removing the thresholds to prevent foot tripping and making them demountable where they need to be used	A	A
R7. Having a not available bathroom in the room	Difficulty reaching the common bathroom	7	0.2	1	1.4	Construction of individual bathrooms in rooms	E	E
R8. Existing common bathroom from away the room	Difficulty reaching the bathroom	7	6	10	420	Designing the location of the bathroom to enable the elderly to access the bathroom easily and quickly	A	A
R9. The dimensions of the room doors are not suitable for stretcher and wheelchair measurements	Emergency response and evacuation difficulties	7	1	10	70	Making the dimensions of the room doors by the size of the stretcher and wheelchair	C	C
R10. The emergency call button is not available	Having difficulty getting help	15	3	10	450	Installing an emergency call button	A	A
R11. Existing no emergency call button of sufficient number and appropriate distance by the size of the room	Having difficulty getting help	15	3	3	135	Installing the call button in sufficient numbers and an easily accessible place	C	C
COMMON AREAS								
A1. The outer door is unprotected and unlocked	Getting lost by escaping from the outer door or being injured by being exposed to various dangers	100	6	6	3600	Establishing a mechanism to ensure that the outer door is protected and locked	A	A
A2. Presence of abrasions and breaks in the floor	Serious injury from falling in case of loss of balance	40	3	10	1200	Making the floor non-slip and suitable material	A	A
A3. Excessive wear on the floor	Injury from falling due to foot tripping	40	3	10	1200	Repairing the worn areas, making the floor material from non-slip and suitable material on the floor	A	A
A4. Lighting does not exist correctly and adequately	Injury from striking objects or falling by tripping over objects on the floor	40	3	10	1200	Installing appropriate quality and quantity lighting fixtures	A	A
A5. The in-room use of furniture is not ergonomic	Injuring from hitting the corners and edges of furniture	15	3	6	270	Making furniture by ergonomic and elderly standards	B	B
A6. The furniture used in the place is made from material that is not suitable for health and the existence of manufacturing defect	Injuring from hitting the corners and edges of furniture	15	3	6	270	Replacing the furniture used in the room with ones made of healthy materials and without manufacturing defects	B	B
A7. The emergency call button is not available	Having difficulty getting help	15	1	6	90	Installing the emergency call button	C	C
A8. Existing no emergency call button of sufficient number and appropriate distance by the size of the room	Having difficulty getting help	7	1	2	14	Installing the emergency call button in sufficient numbers and an easily accessible place	E	E
STAIRS AND ELEVATORS								
S1. The steps are not made from the proper material	Injury or death by falling due to slipped foot	100	10	10	10000	Making the steps of non-slip and user-friendly material	A	A
S2. No precautions against skidding on the steps	Injury or death by falling due to slipped foot	100	10	10	10000	Using anti-skid materials on the steps	A	A
S3. Stair steps being not in the correct height and width	Serious injury or death in the event of a fall due to loss of balance	100	6	10	6000	Making stair dimensions conform to standard dimensions	A	A
S4. Existence not being grab bar on both sides of the stairs	Serious injury or death in the event of a fall due to loss of balance	100	6	10	6000	Installing grab bars on the sides of the stairs	A	A
S5. No being locked door at the beginning of the stair	Serious injury or death in the event of a fall due to loss of balance	100	6	10	6000	Constructing a lockable door at the beginning of the stairs on the floors	A	A
S6. Elevator interior dimensions do not match stretcher and wheelchair size	Emergency response and evacuation difficulties	7	1	10	70	Reconstructing the elevator to match the size of the stretcher and wheelchair	C	C
S7. The timing of the elevator doors not being arranged according to elderly	Difficulty responding to emergencies and evacuation, serious injury, and loss of limb	40	3	10	1200	Adjusting the opening and closing speeds of elevator doors according to the elderly	A	A
S8. Emergency phone or emergency call button not available	Having difficulty getting help	15	3	10	450	Installing the phone or emergency button in the elevator	A	A
S9. No existing generator to run the elevator when cutting electricity	Emergency response and evacuation difficulties	7	1	2	14	Having a ready-to-use generator	E	E
S10. Not existing sufficient and effective lightning the stairs	Serious injury or death in the event of a fall due to loss of balance	100	6	10	6000	Installing appropriate quality and quantity lighting fixtures	A	A

**Table 4:** Risk classes.

Areas	Risk Class					
	A	B	C	D	E	Total
Bathroom	6	2	-	-	1	9
Corridor	4	-	1	3	1	9
Room	6	2	2	-	1	11
Common Area	4	2	1	-	1	8
Stairs and Elevators	8	-	1	-	1	10
Total	28	6	5	3	5	47

In the dataset, probability, frequency, and severity scores which are assigned by experts constitute the input variable values, and traditional ANFIS risk classes constitute the output variable values. The values of the risk factors of each risk and their corresponding risk values were normalized with the 'Maximum-Minimum' method (Eq.10) to increase the accuracy of predictions, and these values were taught to the network.

(10) $$X^{ı}=\frac{X-X_{\min}}{X_{\max}-X_{\min}}$$

where $X_{\min}$ is the minimum value of the dataset, $X_{\max}$ is the maximum value of the dataset, and X is the actual data.

**Figure 3:**
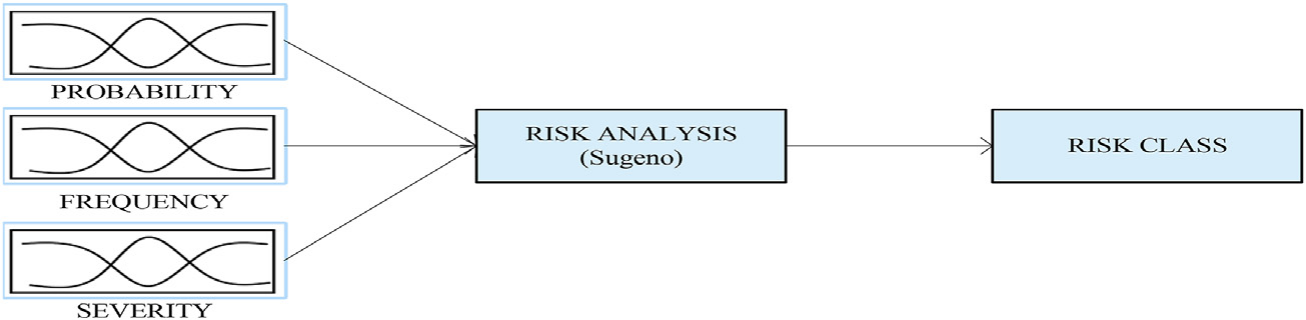
The architecture of the ANFIS model.

The prepared dataset was divided into two subgroups: training and test data. In the study, 80% (47 × 0.80 = 38) of the total data rows were used for training. The remaining data (47 × 0.20 = 9) was used for testing. The ANFIS model, which has three inputs and one output, and the relationship between them is defined by the Sugeno type fuzzy inference system, was created. The architecture of the ANFIS model is shown in Figure 3.

**Figure 4:**
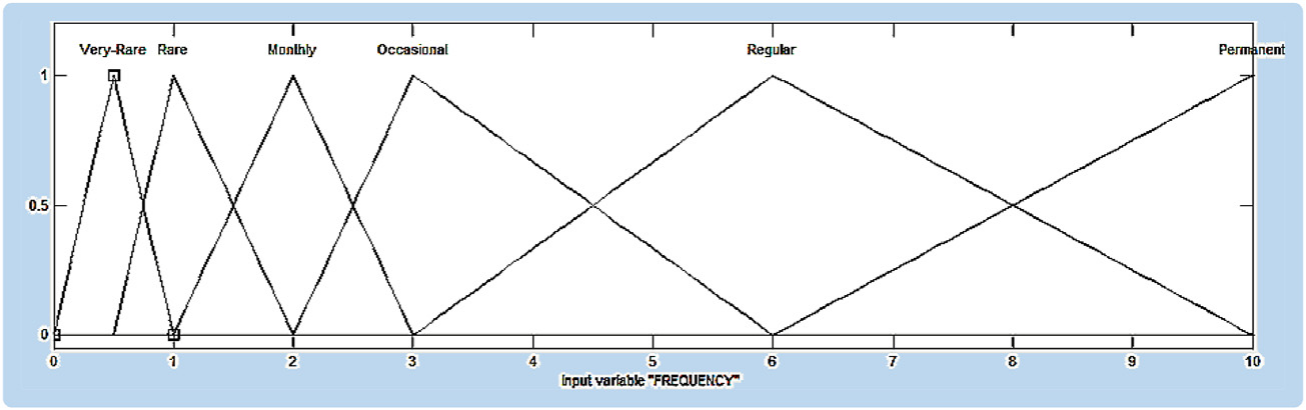
Membership function plots of frequency risk factor.

In the model, seven membership functions for probability and six membership functions for frequency and severity were defined considering the Fine-Kinney method. For the risk value that is the output variable, five fuzzy classes were constituted. For example, the triangular membership function for the frequency input variable is as in Figure 4.

Triangular, trapezoidal, generalized bell, Gaussian, two-sided Gaussian, pi curve, the difference between two sigmoidal functions, the product of two sigmoidal functions membership functions were tried to determine the membership functions of the input variables giving the lowest accuracy error. In addition, models were created by using constant and linear membership functions to determine the membership function of the output variable. For the ANFIS model, 252 if-then rules were created. As an example, the first two and the last two rules are shown below.

*Rule 1: If the probability is Virtually Impossible and the frequency is Very Rare and the severity is Noticeable then the risk class is E.*
*Rule 2: If the probability is Practically Impossible and the frequency is Very Rare and the severity is Noticeable then the risk class is E.*
Rule 251: If the probability is Virtually Impossible and the frequency is Very Rare and the severity is One fatalities-disaster then the risk class is A.
Rule 252: If the probability is Virtually Impossible and the frequency is Very Rare and the severity is Several fatalities- Catastrophe then the risk class is A.

In the study, first of all, the Halving Grid Search (HGS) method was used for a small data set and the hyperparameters affecting the ANFIS method were determined. With the HGS method, it has been determined that the membership function of the input and output variables and the optimization method affect the performance of the model. Then, for the whole data set, the optimum values were found by combining all the determined hyperparameters. In the study, all possible grid searches were performed using 32 combinations for 3 hyperparameters determined by HGS of the ANFIS method (Table 5). The Pi curve membership function (pimf) gives the lowest error (1.628e-06) among the eight membership functions selected for this purpose. Error-values of membership functions are as in Table 5.

**Table 5:** Membership function and error value.

Input MF type	Error			
	*Train Optimization Algorithm*			
	Hybrid		Backpropagation	
	*Output MF Type*			
	Constant	Linear	Constant	Linear
Triangular MF (trimf)	3.191e-06	2.431e-06	0.162	0.053
Trapezoidal MF (trapmf)	3.288e-06	2.465e-06	0.162	0.052
Generalized bell MF (gbellmf)	4.930e-06	3.479e-06	0.526	0.216
Gaussian MF (gaussmf)	4.464e-06	3.193e-06	0.136	0.126
Two-sided Gaussian MF (gauss2mf)	1.912e-06	3.193e-06	0.126	0.117
Pi curve MF (pimf)	2.164e-06	***1.628e-06***	0.129	0.066
Difference between two sigmoidal functions MF (dsigmf)	3.623e-06	2.604e-06	0.162	0.100
Product of two sigmoidal functions MF (psigmf)	3.623e-06	2.605e-06	0.109	0.099

MF: Membership function

**Figure 5:**
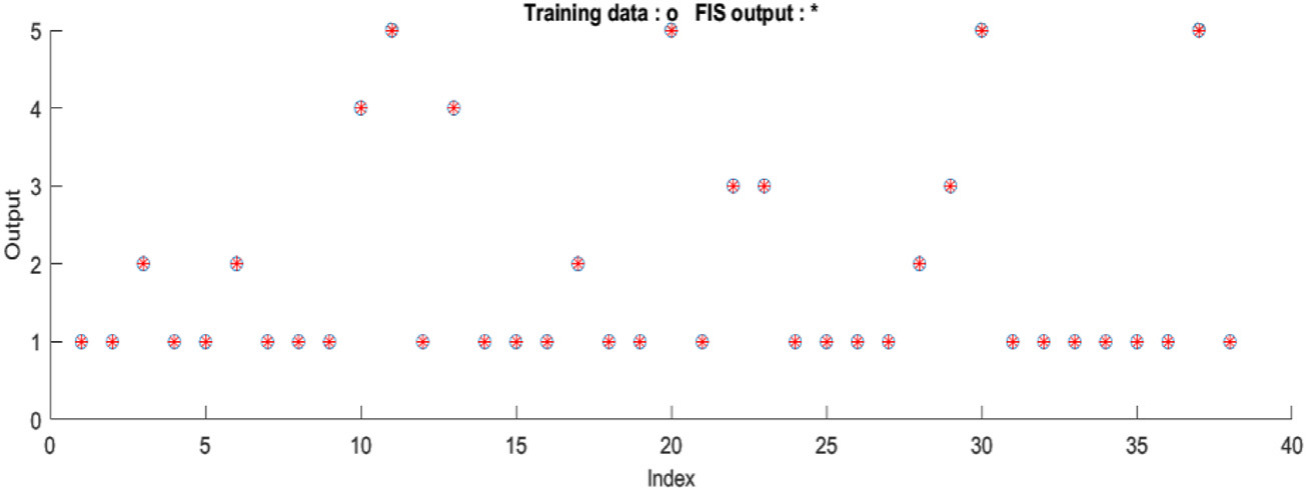
Test plot of training output.

All combinations of the model were stopped after 1000 iterations and the error tolerance was set to 0. Pi curve input membership function, linear output membership function, and hybrid training optimization algorithm that combines least-square estimator and gradient descent method provided the lowest error rate (1.628e-06). After the model was trained, the training and testing dataset were tested. Figure 5 shows that all of the training data were correctly predicted.

**Figure 6:**
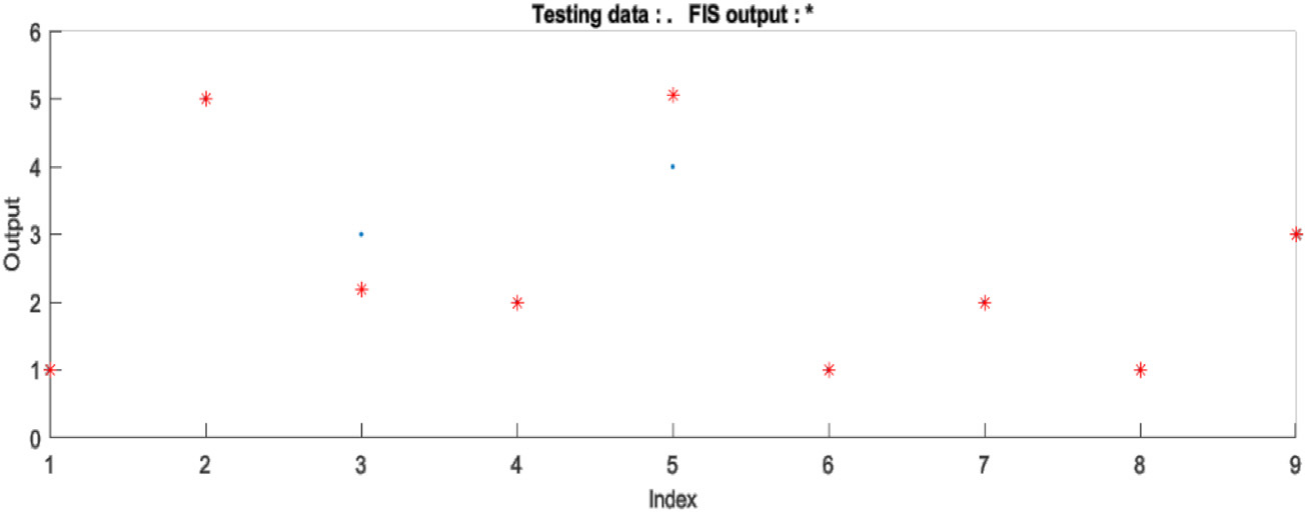
Test plot of testing output.

In Figure 6, it is seen that the 3rd risk class that should be assigned to Class C has been assigned to Class B. Likewise, the 5th risk class has been assigned to Class E instead of Class D.

The MATLAB 2014A ANFIS toolbox is used for ANFIS applications.

The classes of risks determined by the hybrid method based on Fine- Kinney, and ANFIS were compared with those determined by the traditional Fine-Kinney method. The confusion matrix, which includes the results of both methods, was obtained (Table 6).

**Table 6:** Confusion matrix for risk classes.

ANFIS Fine-Kinney Class							
	Traditional Fine-Kinney Method Class					Classification Overall	User's Accuracy (Precision) (%)
	A	B	C	D	E		
A	28	0	0	0	0	28	100
B	0	6	1	0	0	7	85.71
C	0	0	4	0	0	4	100
D	0	0	0	2	0	2	100
E	0	0	0	1	5	6	83.33
Truth Overall	28	6	5	3	5	47	
Producer's Accuracy (Recall) (%)	100	100	80	66.67	100		

As shown in Tables 6 and 45 of the 47 risks were classified in the same class both traditional and the developed hybrid Fine-Kinney methods. Risks classified differently are C4 and C5 (Table 3). While the C5 risk should be in the C class according to the traditional Fine-Kinney method, it has been assigned to the B risk class in the hybrid method due to the high severity and frequency values. In addition, the C4 risk, which has a risk score of 21 according to the traditional Fine-Kinney method and should be assigned to the D risk class, has been assigned to the E class. On the other hand, the C8 risk, which has a risk score of 20 and has a lower priority than the C4 risk according to the traditional Fine-Kinney method, is assigned to the D class in the hybrid method. This is because the severity value of the C8 risk is very high.

The overall accuracy of proposed model is determined as 95.745% from Eq. (8). Similarly, the Fleiss' kappa coefficient is calculated as 0.929 by Eq. (9). In order to evaluate the prediction accuracy of the developed hybrid method, hybrid methods using ANN and Fuzzy logic instead of ANFIS method were applied to the same spatial risk values. While the developed hybrid Fine Kinney and ANFIS method predicts only 2 of the risk classes incorrectly; The hybrid Fine Kinney-ANN method and the hybrid Fine Kinney-fuzzy method predicts 3 and 5 incorrect classes, respectively (Table 7).

**Table 7:** Confusion matrix for hybrid Fine Kinney- ANN and hybrid Fine Kinney-fuzzy methods.

	Traditional Fine-Kinney Method Class					
ANN (Fuzzy) Fine-Kinney Class		A	B	C	D	E
	A	28 (27)	0 (0)	0 (0)	0 (0)	0 (0)
	B	0 (0)	6 (5)	1 (0)	0 (0)	0 (0)
	C	0 (0)	0 (0)	3 (3)	1 (0)	0 (0)
	D	1 (1)	0 (1)	0 (1)	2 (3)	0 (1)
	E	0 (1)	0 (0)	0 (0)	0 (0)	5 (4)

The fuzzy model's classes are shown in brackets in this table.

The results of the performance criteria are given in Table 8. These performance values show that the developed novel hybrid method has the highest ability to accurately predict risk classes. In other words, the results obtained by the hybrid Fine-Kinney and ANFIS method are similar to those found by the traditional Fine-Kinney method.

**Table 8:** Comparison of the hybrid methods.

Performance Criterion	Method		
	Fine-Kinney and ANFIS methods	Fine Kinney and ANN methods	Fine Kinney and Fuzzy methods
Overall accuracy	95.745%	93.617%	89.362%
Kappa coefficient	0.929	0.892	0.824

## 5 Conclusion

It is aimed to develop a novel hybrid risk assessment method using Fine-Kinney and ANFIS methods to eliminate the shortcomings of the traditional Fine Kinney method in this study. The FIS structure of ANFIS enables experts to evaluate risk by verbal expressions instead of a crisp number. Since the risk classes taught in the ANFIS model are determined by the opinions of many experts, the prediction of a new risk class can be defined by only an expert, that is, the dependency on experts reduce. While developing the ANFIS model, it is possible to evaluate the risk factors in different degrees of importance. Because the class of the newly occurring risk can be easily and correctly predicted with the ANFIS model learned without applying the Fine Kinney method steps. The novel method helps experts to reduce time consumption and labor intensiveness. As the risks are evaluated more effectively and quickly, correct corrective and preventive actions can be developed and implemented rapidly.

The elimination or reduction of risks will positively affect the quality of life of the elderly living in nursing homes. The risks to which corrective and preventive actions will be first applied are determined according to the results of the spatial risk analysis. The results are also used in the planning of the spaces, selecting decoration and furniture in new nursing homes to be constructed.

The contributions of the new hybrid method can be summarized as follows:

• The hybrid method can assist experts in improving the effectiveness of risk analysis and determining the risk class of spatial in a short time.
• It can also be used in the spatial risk assessment of places, such as schools, hospitals, and factories.

## Declarations

### Author contribution statement

Didem ÇAPKUR YILMAZ, Dr; Zerrin Funda ÜRÜK, Dr: Conceived and designed the experiments.

Semra BORAN, Prof. Dr; Seda Hatice GÖKLER, Dr: Conceived and designed the experiments; Performed the experiments; Analyzed and interpreted the data; Contributed reagents, materials, analysis tools or data; Wrote the paper.

### Funding statement

This research did not receive any specific grant from funding agencies in the public, commercial, or not-for-profit sectors.

### Data availability statement

Data included in article/supp. material/referenced in article.

### Declaration of interest's statement

The authors declare no conflict of interest.

### Additional information

No additional information is available for this paper.
